# Diagnosis of thyroid disease using deep convolutional neural network models applied to thyroid scintigraphy images: a multicenter study

**DOI:** 10.3389/fendo.2023.1224191

**Published:** 2023-08-11

**Authors:** Huayi Zhao, Chenxi Zheng, Huihui Zhang, Maohua Rao, Yixuan Li, Danzhou Fang, Jiahui Huang, Wenqian Zhang, Gengbiao Yuan

**Affiliations:** Department of Nuclear Medicine, The Second Affiliated Hospital of Chongqing Medical University, Chong Qing, China

**Keywords:** deep convolutional neural network, thyroid scintigraphy, artificial intelligence, thyroid disease, nuclear medicine physicians

## Abstract

**Objectives:**

The aim of this study was to improve the diagnostic performance of nuclear medicine physicians using a deep convolutional neural network (DCNN) model and validate the results with two multicenter datasets for thyroid disease by analyzing clinical single-photon emission computed tomography (SPECT) image data.

**Methods:**

In this multicenter retrospective study, 3194 SPECT thyroid images were collected for model training (n=2067), internal validation (n=514) and external validation (n=613). First, four pretrained DCNN models (AlexNet, ShuffleNetV2, MobileNetV3 and ResNet-34) for were tested multiple medical image classification of thyroid disease types (i.e., Graves’ disease, subacute thyroiditis, thyroid tumor and normal thyroid). The best performing model was then subjected to fivefold cross-validation to further assess its performance, and the diagnostic performance of this model was compared with that of junior and senior nuclear medicine physicians. Finally, class-specific attentional regions were visualized with attention heatmaps using gradient-weighted class activation mapping.

**Results:**

Each of the four pretrained neural networks attained an overall accuracy of more than 0.85 for the classification of SPECT thyroid images. The improved ResNet-34 model performed best, with an accuracy of 0.944. For the internal validation set, the ResNet-34 model showed higher accuracy (*p* < 0.001) when compared to that of the senior nuclear medicine physician, with an improvement of nearly 10%. Our model achieved an overall accuracy of 0.931 for the external dataset, a significantly higher accuracy than that of the senior physician (0.931 vs. 0.868, *p* < 0.001).

**Conclusion:**

The DCNN-based model performed well in terms of diagnosing thyroid scintillation images. The DCNN model showed higher sensitivity and greater specificity in identifying Graves’ disease, subacute thyroiditis, and thyroid tumors compared to those of nuclear medicine physicians, illustrating the feasibility of deep learning models to improve the diagnostic efficiency for assisting clinicians.

## Introduction

In recent years, the worldwide incidence rate of thyroid disease has increased (1, 2), making it the second-most prevalent disease in the field of endocrinology (3, 4). In clinical practice, pertechnetate (^99m^TcO_4_^-^) thyroid scintigraphy is currently the primary method of observing the position, shape, size, and functional status of the thyroid gland and plays a significant guiding role in the treatment of various thyroid diseases (5, 6). The insidious onset and lack of specificity in most thyroid diseases often lead to misdiagnosis. Thus, the best treatment opportunity is often missed, even when clinical symptoms appear, impacting patient prognosis. Many studies have demonstrated that thyroid scintigraphy is an effective method for differentiating Graves’ disease, thyroiditis, and thyroid tumors (7, 8). In addition, the interpretation of thyroid scintigraphy findings relies heavily on the expertise of nuclear medicine physicians. The final diagnosis results are easily limited by physicians’ experience and subjective factors, which can lead to misdiagnosis or affect the accuracy rate. Therefore, the use of deep learning technology to assist clinicians in the high-precision diagnosis of thyroid diseases is of great clinical significance.

DCNN is a deep learning structure that retains spatial correlations in two-dimensional data and is extensively employed for artificial intelligence (AI) applications owing to its powerful feature extraction capabilities (9). Recently, deep learning algorithms based on DCNNs have shown great potential for medical image recognition, achieving exceptional diagnostic accuracy and efficiency in interpreting medical images (10–14). DCNNs have been widely used to evaluate thyroid lesions on ultrasound, computed tomography (CT), and magnetic resonance (MR) images, providing supportive advice for clinical diagnosis (15, 16). In the field of ultrasound diagnosis, differentiating between benign and malignant thyroid nodules typically yields a diagnostic accuracy range of 85% to 90%. Li et al. (17) developed a DCNN model trained with hundreds of thousands of thyroid ultrasound images. The model achieved recognition accuracies ranging from 85.7% to 88.9% across three validation sets. Another study conducted by Qi et al. (18) utilized a dataset of over 4,000 ultrasound images to develop a deep learning model specifically tailored for localizing and evaluating thyroid cancer nodules in preoperative ultrasound images. The model consistently achieved an accuracy exceeding 85% across multiple test sets. In terms of CT diagnosis, Zhang et al. (15) constructed a diagnostic model for thyroid diseases utilizing a dataset containing over 2,000 CT images of the thyroid gland, achieving an impressive accuracy rate of 94.2%. Furthermore, DCNNs can achieve performance that meets or exceeds that of human experts in multiple medical image classification or detection tasks (19). However, while there are many studies on imaging modalities, few studies have utilized algorithms based on deep learning to automatically categorize thyroid scintigraphy images.

In this study, we used thyroid scintigraphy images to classify and diagnose Graves’ disease, subacute thyroiditis, thyroid tumors, and normal subjects using DCNN models and then compared the results of those methods with those of nuclear medicine physicians. Finally, we validated the DCNN models with independent internal and external datasets. The accurate differential diagnosis of thyroid tumor images is a novel aspect of this study.

## Methods

### Study cohort

This study was reviewed and approved by the Ethics Committee of the Second Affiliated Hospital of Chongqing Medical University and was conducted in accordance with the Helsinki Declaration. Since this study was a retrospective study with minimal risk, the requirement for informed patient consent was waived.

In this study, we conducted a retrospective, multicenter diagnostic study on SPECT thyroid scintigraphy images from three hospitals in Chongqing. We retrospectively collected ^99m^TcO_4_^-^ thyroid images from cases at the Second Affiliated Hospital of Chongqing Medical University (Center 1), Southwest Hospital (Center 2), and Chongqing People’s Hospital (Center 3) from January 2013 to July 2022. Ultimately, the images collected by Center 1 were used for training and internal validation of the DCNN models. The external validation set consisted of the images collected in Centers 2 and 3.

All collected case images met the specified criteria: 1. The diagnoses were verified through clinical history and ancillary tests, including radioiodine uptake tests, thyroid function tests, and ultrasonography. For many cases, the diagnoses were confirmed after follow-up treatment. 2. Patients with thyroid tumors underwent cytological examination or pathological histological examination. 3. The final clinical diagnosis and all thyroid scintigraphy imaging results were in agreement. The exclusion criteria were as follows: 1. Patients who underwent semi/total thyroidectomy. 2. Incomplete image data. 3. Poor-quality SPECT images.

### Classification criteria

**Figure 1:**
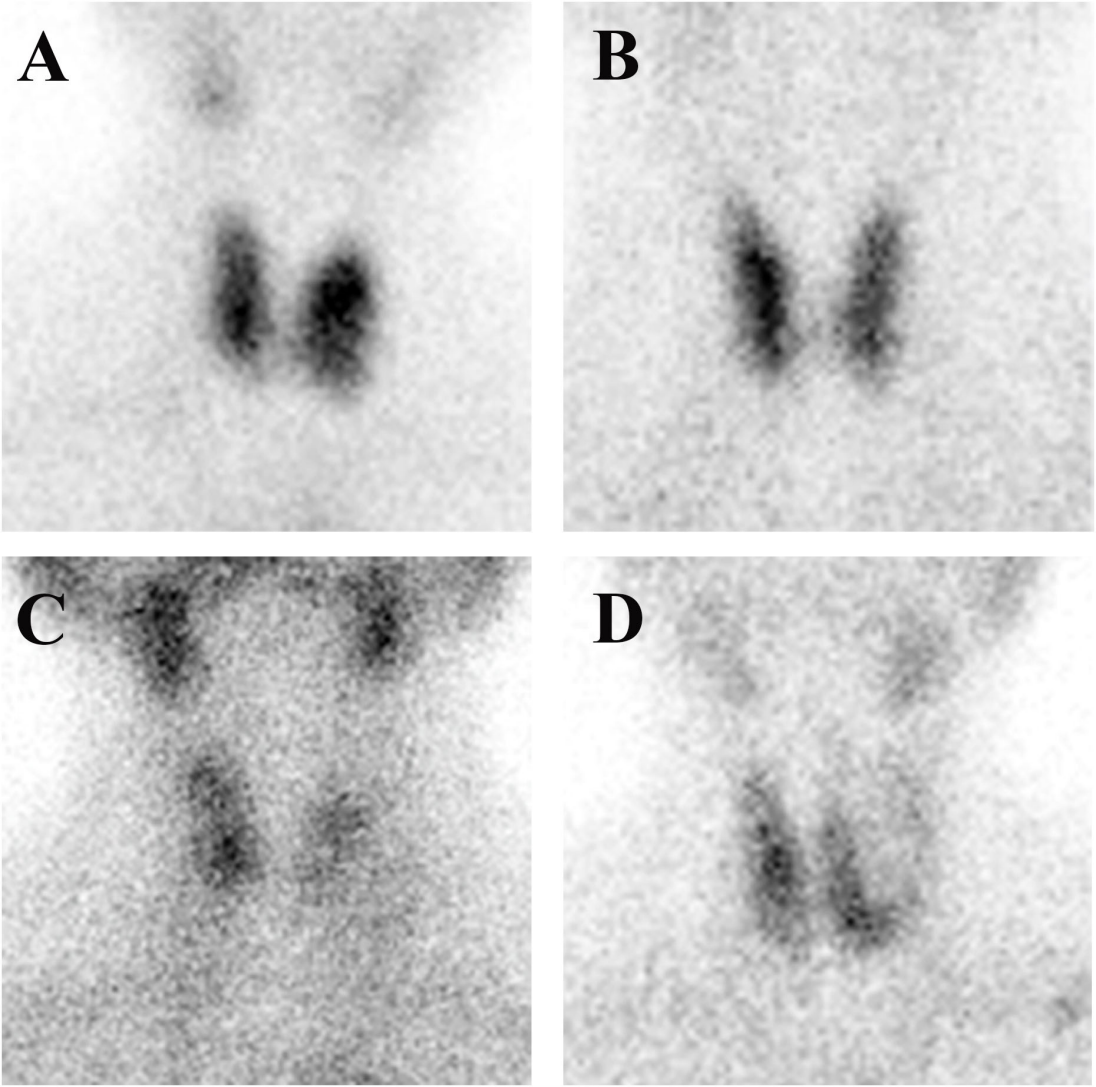
Four different thyroid images: **(A)** Graves’ disease; **(B)** normal thyroid; **(C)** subacute thyroiditis; and **(D)** thyroid tumor.

Samples of the four types of thyroid images are shown in **Figure 1**. Graves’ disease typically manifests as an enlarged gland with a diffuse concentration of contrast uptake and distribution. The normal thyroid gland has a normal location and size, regular morphology, and a generally uniform distribution of contrast medium in both lobes. Subacute thyroiditis tends to present early in the course of the disease with areas of limited or diffuse reduced radionuclide uptake. Thyroid tumors can be classified as either benign or malignant. Benign tumors are typically identified as hot or warm nodules, while malignant tumors are predominantly cold nodules that may invade surrounding and contralateral normal tissues. The thyroid tumor samples include benign tumors (thyroid adenoma and thyroid cystadenoma) and malignant thyroid cancer (papillary carcinoma, follicular carcinoma, medullary carcinoma, and undifferentiated carcinoma). A detailed description of the differences in image patterns for each disease is provided in the **Supplementary Material**.

### Imaging protocols

Thyroid scintigraphy was performed at three different hospitals in accordance with clinical guidelines and the manufacturer’s recommended parameters. The images were acquired using a Millennium VG scanner, a Symbia T6 SPECT/CT, and a Symbia Intevo Bold scanner. The subsequent image acquisition was carried out by following the instructions for the SPECT equipment provided by Siemens and GE Healthcare.

### Data preprocessing

All thyroid SPECT images extracted from the thyroid imaging databases of the three hospitals were in JPG format. To obtain high-quality SPECT images, all thyroid images were cleaned. All images were screened by two nuclear medicine doctors with ≥ 5 years of SPECT imaging experience. We randomly divided all thyroid images from Center 1 into two groups, a training set and an internal validation set, with a ratio of approximately 8:2. Detailed information on data preprocessing is provided in the **Supplementary Material**.

### Network architecture

**Figure 2:**
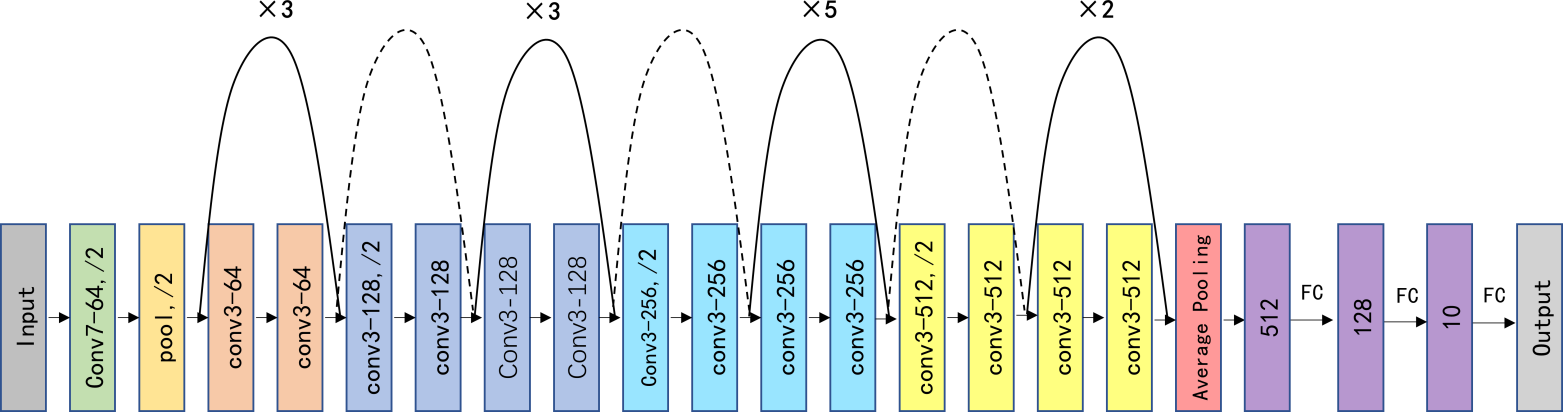
Schematic visualization of the improved ResNet-34 model architecture. Conv, pool, and FC represent the convolution kernel, pooling, and fully connected layers, respectively. “×3/5/2” indicates the number of times the structure is repeated; “/2” indicates the corresponding step size, “Conv7-64” means a convolutional core size of 7 × 7 and a filter number of 64 for the input and output, and the dashed lines indicate different input and output sizes.

In this study, we appropriately modified the fully connected (FC) layer in ResNet-34 and compared this modified model with three pretrained AI models, including AlexNet (20), ShuffleNetV2 (21), MobileNetV3 (22), and ResNet-34 (23). The structure of our improved deep convolutional neural network (ResNet-34) is depicted in **Figure 2**. The whole network structure consists of a dual-stage process. In the first stage of the process, the main features are extracted from the input SPECT images using the ResNet-34 architecture, which is currently one of the most widely used deep residual networks. The final FC layer in the original ResNet-34 was removed. The four modules included in ResNet-34 can extract a total of 512 features from low to high levels. Then, all 512 features were processed using two FC layers with leaky rectified linear unit (ReLU) activation and one FC layer with softmax activation (24) to calculate and output the probability of a patient having a studied thyroid diseases. The ReLU activation function introduces nonlinearity to enhance the expressive capacity of the DCNN. The shortcut connection facilitates a direct transmission of input information to output, ensuring the integrity of information transmission. To enhance the generalizability of the model and minimize the risk of overfitting, we incorporated dropout into the first FC layer, with a dropout probability of 0.8 (25).

### Training and evaluation methods

Cross-entropy (26) is a commonly used loss function. It is widely used in classification tasks because it allows training to converge quickly. As there was no category imbalance in our data, we utilized the cross-entropy loss function to optimize the model. The adaptive moment estimation (Adam) optimizer, the most stable optimizer in DCNN construction, was employed with an attenuation weight of 1e-4 to update the weight parameters during the training process. The initial learning rate was set to 2.5 × 10 ^−5^ and was gradually updated using the stochastic gradient descent algorithm during fine-tuning. Specifically, the learning rate was reduced to one-tenth of the original rate after every 80 batches. We set the batch size to 12 and the maximum number of iterations to 200. To ensure the rigorous evaluation of all datasets included in this study and minimize the potential variance caused by limited datasets, we conducted 5-fold cross-validation on the model with the highest accuracy.

Thyroid images from both validation sets were provided to one junior nuclear medicine physician with 2 years of experience (L.Y.X.) and one senior nuclear medicine physician with 5 years of experience (Z.C.X.). These physicians were blinded to the patient information and classification results. Their diagnostic performance was then compared with that of the best DCNN model. In addition, all neural network models were implemented on the PyTorch platform and trained and tested with the same computing conditions. Computing was performed using hardware with an NVIDIA GeForce RTX 3080 Ti GPU. See the **Supplementary Material** for details on the operating environment.

### Feature visualization

Currently, DCNNs are considered uninterpretable and are still in the ‘black box’ stage (27, 28). To better interpret the predictions of our DCNN models, we utilized gradient-weighted class activation mapping (Grad-CAM) to generate heatmaps (29). The Grad-CAM method was chosen to interpret the network predictions and provide better insights into the reasoning behind them. We extracted feature weights from the last convolutional layer of the DCNN model to build the class activation map.

### Statistical analysis

To compare the performance of the deep learning model with that of nuclear medicine physicians, we constructed receiver operating characteristic (ROC) curves. The DeLong test was used to determine the significant differences in the area under the receiver operating characteristic curve (AUC) between the various diagnostic methods. We reported accuracy, sensitivity, specificity, negative predictive value (NPV) and positive predictive values (PPV) with 95% confidence intervals (CIs) for both the deep learning models and nuclear medicine physicians, as well as κ values and F1 scores. The differences in accuracy between the DCNN model and clinical physicians were compared using the chi-squared test. The statistical analyses were performed using SPSS software (version 26.0), MedCalc (version 20.218), and R (version 4.2.2) statistical software, with *P* < 0.05 considered statistically significant.

## Results

### Study population

**Figure 3:**
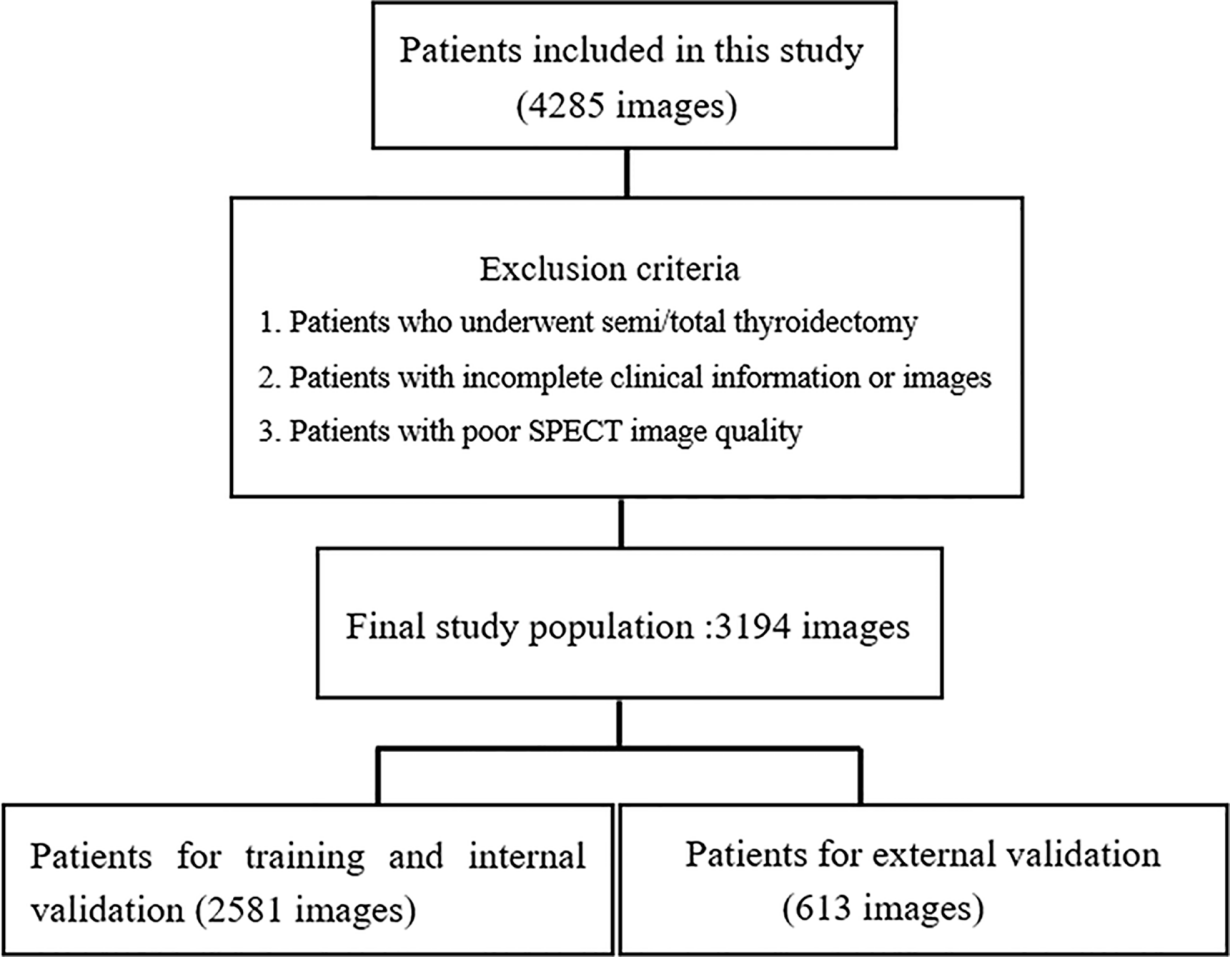
Flowchart of the exclusion criteria for the final study population. A total of 3194 patients from three hospitals participated in the study. The images collected from 2581 patients from one hospital were used for training and internal validation, and the images from 613 patients from the other two hospital were used for external validation.

A total of 4285 SPECT images were retrospectively collected for this study. Of these images, 1091 images were excluded based on our inclusion and exclusion criteria. Therefore, 3194 SPECT images were eventually included in this study (**Figure 3**). Among the 3194 patients (3194 images) included in the study, 2329 (72.92%) were female and 865 (27.08%) were male, with an average age of 45.00 ± 14.50. **Table 1** shows the fundamental clinical characteristics of the patient cohort under investigation in this study.

**Table 1:** Baseline characters of patients.

Variables	All patients	Training cohort	Internal Validation	External Validation
Number of	3194	2067 (64.72%)	514 (16.09%)	613 (19.19%)
Patients (%)				
Age	45.00 ± 14.50	45.48 ± 14.31	46.05 ± 15.82	42.49 ± 13.70
Gender (%)				
Male	865 (27.08%)	564 (27.29%)	148 (28.79%)	153 (24.96%)
Female	2329 (72.92%)	1503 (72.71%)	366 (71.21%)	460 (75.04%)
Disease type (%)				
Graves’ disease	808	520 (25.16%)	130 (25.29%)	158 (25.77%)
Normality	742	484 (23.42%)	120 (23.35%)	138 (22.51%)
SAT	826	531 (25.69%)	132 (25.68%)	163 (26.59%)
Tumor	818	532 (25.74%)	132 (25.68%)	154 (25.12%)

Qualitative variables are in n (%), and quantitative variables are in mean ± SD when appropriate.

### Preliminary DCNN model predictions

**Figure 4:**
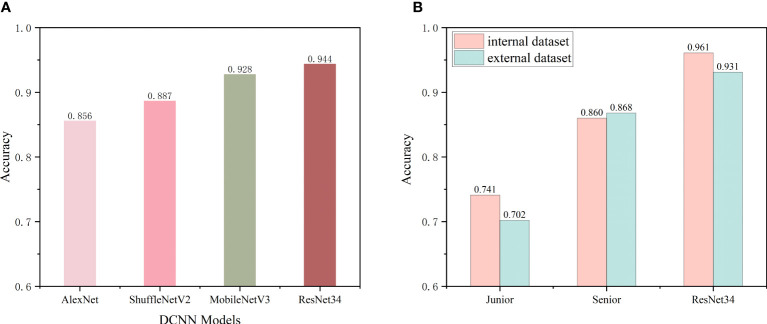
**(A)** Preliminary predictive performance of the four DCNN network models with the internal datasets. **(B)** Performance of junior and senior nuclear medicine physicians and the ResNet-34 model with the internal and external validation sets.

The internal dataset was randomly split into a training set and a validation set at a ratio of 8:2. The improved ResNet-34 model and the other three DCNN models (AlexNet, ShuffleNetV2 and MobileNetV3) were used to make initial predictions with the internal validation dataset. The metrics for the classifications using the four DCNN models with the internal validation set are listed in **Table 2**. These four DCNN models achieved good performance in terms of thyroid disease identification with the validation set. Our improved model performed better than the other models. The overall accuracies of AlexNet, ShuffleNetV2, MobileNetV3 and ResNet-34 with the internal validation set were 0.856, 0.887, 0.928 and 0.944, respectively (**Figure 4A**), and the κ values were 0.808, 0.850, 0.904 and 0.925, respectively (**Table 2**). The ROC curves for these four DCNN models for the diagnostic internal dataset are shown in **Supplementary Figure S3**. Notably, the AUC of the ResNet-34 model was 0.992, indicating its superior performance compared to that of the other models.

**Table 2:** Performance of each model on the internal validation set.

model	class	Recall	Specificity	Precision	NPV	AUC	F1	κ value
AlexNet	Graves’ disease	92.3	99.0	96.8	97.4	0.996	0.949	0.808
	Normality	92.5	89.6	73.0	97.5	0.970	0.816	
	SAT	90.9	98.2	94.5	96.9	0.992	0.927	
	Tumor	67.4	94.2	80.2	89.3	0.930	0.733	
ShuffleNetV2	Graves’ disease	98.5	97.4	92.8	99.5	0.995	0.955	0.850
	Normality	95.0	91.9	78.1	98.4	0.964	0.857	
	SAT	93.2	99.0	96.9	97.7	0.994	0.950	
	Tumor	68.9	96.9	88.4	90.0	0.937	0.775	
MobileNetV3	Graves’ disease	100.0	98.7	96.3	100.0	0.999	0.981	0.904
	Normality	95.0	96.2	88.4	98.4	0.990	0.916	
	SAT	93.9	98.2	94.7	97.9	0.997	0.943	
	Tumor	82.6	97.4	91.6	94.2	0.973	0.869	
ResNet34	Graves’ disease	97.7	99.0	96.9	99.2	0.997	0.973	0.925
	Normality	96.7	97.2	91.3	99.0	0.991	0.939	
	SAT	93.9	99.2	97.6	97.9	0.992	0.958	
	Tumor	89.4	97.1	91.5	96.4	0.980	0.904	

SAT, Subacute thyroiditis; AUC, the area under the curve; κ value, Fleiss’s κ value; NPV, negative predictive value; PPV, positive predictive value.

The values below indicate 95% CIs.

### Cross-validation

A 5-fold cross-validation was performed with an internal dataset to quantitatively evaluate the classification performance of the improved ResNet-34 model. The results are shown in **Table 3**. The mean classification accuracy, sensitivity and specificity of our model on the 5-fold cross-validation were 94.8%, 94.8% and 98.3%, respectively, and the standard deviation did not exceed 0.01, demonstrating the excellent classification performance and robustness of the improved ResNet-34 model for thyroid SPECT images.

**Table 3:** The result of the five-fold cross-validation.

Fold	Accuracy	Recall	Specificity	PPV	NPV	AUC
1	94.6%	94.6%	98.2%	94.5%	98.2%	0.990
2	95.2%	95.2%	98.4%	95.1%	98.4%	0.990
3	94.4%	94.5%	98.1%	94.5%	98.2%	0.994
4	96.1%	96.1%	98.7%	96.1%	98.7%	0.993
5	93.6%	93.6%	97.9%	93.7%	97.9%	0.989
Mean ± std	94.8% ± 0.009	94.8% ± 0.009	98.3% ± 0.003	94.8% ± 0.009	98.3% ± 0.003	0.991 ± 0.002

### Comparison of the diagnostic performance between nuclear medicine physicians and the best DCNN model

As the improved ResNet-34 performed better than the other models, we compared the diagnostic performance of this model with that of nuclear medicine physicians. Two nuclear medicine physicians, who were blinded to the cytology data and biochemical indicators, performed differential diagnoses using SPECT images from the internal and external validation sets. **Figure 4B** shows the classification accuracies of the model and the nuclear medicine physicians with the two independent validation datasets. The recognition accuracies for the junior and senior physicians and the model were 0.741, 0.860, and 0.961 with the internal validation set and 0.702, 0.868, and 0.931 with the external validation set, respectively. These findings emphasize that the senior nuclear medicine physician outperformed the junior physician with the internal and external validation sets by more than 10% (*p*<0.001). Additionally, our model achieved higher accuracies in identifying SPECT thyroid images with both validation sets compared to those of the experienced physician (*p*<0.001), improvements of approximately 6% - 10%. The confusion matrices of the two validation datasets using ResNet-34 are shown in **Supplementary Figure S2**.

**Tables 4**, **5** show the performance of the DCNN model and the junior and senior nuclear medicine physicians for each classification with the internal and external validation sets, respectively. The DCNN achieved the highest overall accuracy of 0.961 when classifying four common thyroid scintigram images in the internal validation set, whereas the overall accuracy dropped to 0.931 with the external validation set. After applying the ROC, the DCNN for the diagnosis of three thyroid diseases achieved a considerable performance, with an AUC of 0.993 for Graves’ disease, 0.994 for normal thyroid, 0.999 for subacute thyroiditis, and 0.983 for thyroid tumor with the internal validation set. Accordingly, satisfactory results of 0.969, 0.985, 0.996, and 0.981, respectively, were obtained using this model with the external validation set.

**Table 4:** Performance metrics of the ResNet34 model with junior and senior nuclear medicine physicians on the internal validation set.

	ResNet34				Junior				Senior			
	Graves’ disease	Normality	SAT	Tumor	Graves’ disease	Normality	SAT	Tumor	Graves’ disease	Normality	SAT	Tumor
Recall (%)	97.7 93.4-99.2	95.8 90.6-98.2	100.0 97.2-100.0	90.9 84.8-94.7	84.6 77.4-89.8	75.0 66.6-81.9	76.5 68.6-82.9	60.6 52.1-68.5	93.8 88.3-96.8	81.7 73.8-87.6	94.7 89.5-97.4	73.5 65.4-80.3
Specificity (%)	99.5 98.1-99.9	98.0 96.0-99.0	99.0 97.3-99.6	98.4 96.6-99.3	91.4 88.2-93.8	89.6 86.2-92.2	96.6 94.3-98.0	88.0 84.3-90.8	99.2 97.7-99.7	96.7 94.4-98.1	87.7 84.0-90.6	97.6 95.6-98.8
PPV (%)	98.4 94.5-99.6	93.5 87.7-96.7	97.1 92.7-98.9	95.2 90.0-97.8	76.9 69.4-83.1	68.7 60.3-76.0	88.6 81.5-93.2	63.5 54.8-71.4	97.6 93.2-99.2	88.3 81.0-93.0	72.7 65.6-78.8	91.5 84.6-95.5
NPV (%)	99.2 97.7-99.7	98.7 97.0-99.5	100.0 99.0-100.0	96.9 94.7-98.2	94.6 91.8-96.5	92.2 89.0-94.5	92.3 89.2-94.5	86.6 82.8-89.6	97.9 96.0-99.0	94.5 91.9-96.4	98.0 95.8-99.0	91.4 88.3-93.8
AUC	0.993 0.982- 0.998	0.994 0.983-0.999	0.999 0.992-1.000	0.983 0.968-0.993	0.880 0.849-0.907	0.823 0.787-0.855	0.866 0.833-0.894	0.743 0.703-0.780	0.965 0.946-0.979	0.892 0.862-0.914	0.912 0.884-0.935	0.856 0.822-0.885
F1	0.981	0.947	0.985	0.931	0.806	0.720	0.821	0.620	0.957	0.848	0.822	0.815
K value	0.948				0.655				0.813			

SAT, Subacute thyroiditis; AUC, the area under the curve; κ value, Fleiss’s κ value; NPV, negative predictive value; PPV, positive predictive value.

The values below indicate 95% CIs.

**Table 5:** Performance metrics of the ResNet34 model with junior and senior nuclear medicine physicians on the external validation set.

	ResNet34				Junior				Senior			
	Graves’ disease	Normality	SAT	Tumor	Graves’ disease	Normality	SAT	Tumor	Graves’ disease	Normality	SAT	Tumor
Recall (%)	88.0 82.0-92.2	94.2 89.0-97.0	97.5 93.9-99.0	92.9 87.7-96.0	70.3 62.7-76.8	75.4 67.6-81.8	82.8 76.3-87.8	51.9 44.1-59.7	88.6 82.7-92.7	94.2 89.0-97.0	92.0 86.8-95.3	72.7 65.2-79.1
Specificity (%)	100.0 99.2-100.0	94.5 92.1-96.2	99.8 98.8-100.0	96.7 94.7-98.0	93.8 91.2-95.7	78.1 74.2-81.6	96.4 94.3-97.8	92.4 89.6-94.5	96.9 94.9-98.2	92.4 89.7-94.5	97.3 95.4-98.5	95.9 93.6-97.3
PPV (%)	100.0 97.3-100.0	83.3 76.7-88.4	99.4 96.5-100.0	90.5 84.9-94.2	79.9 72.4-85.7	50.0 43.3-56.7	89.4 83.5-93.4	69.6 60.6-77.2	90.9 85.3-94.5	78.3 71.4-83.9	92.6 87.5-95.7	85.5 78.5-90.5
NPV (%)	96.0 93.8-97.4	98.2 96.6-99.1	99.1 97.8-99.7	97.6 95.7-98.6	90.1 87.1-92.5	91.6 88.5-93.9	93.9 91.4-95.8	85.1 81.7-88.0	96.1 93.9-97.5	98.2 96.5-99.1	97.1 95.1-98.3	91.3 88.4-93.5
AUC	0.969 0.952-0.981	0.985 0.972-0.993	0.996 0.988-0.999	0.981 0.966-0.990	0.820 0.788-0.850	0.767 0.732-0.800	0.896 0.869-0.919	0.722 0.684-0.757	0.928 0.904-0.947	0.933 0.910-0.952	0.947 0.926-0.963	0.843 0.812-0.871
F1	0.936	0.884	0.985	0.917	0.747	0.601	0.860	0.595	0.897	0.855	0.923	0.786
K value	0.909				0.603				0.824			

SAT, Subacute thyroiditis; AUC, the area under the curve; κ value, Fleiss’s κ value; NPV, negative predictive value; PPV, positive predictive value.

The values below indicate 95% CIs.

**Figure 5:**
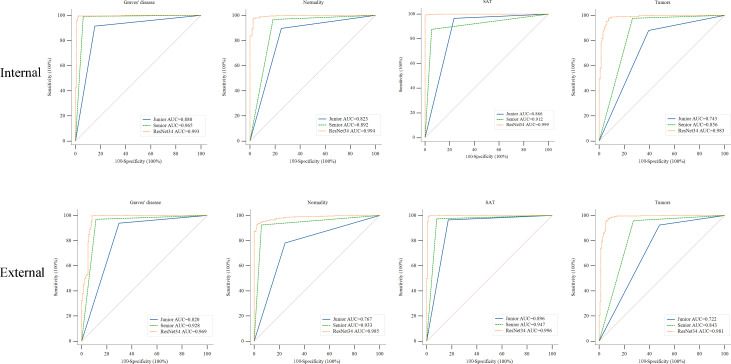
ROC curves of the ResNet-34 model and the junior and senior nuclear medicine physicians for differentiating thyroid diseases. The yellow, blue and green lines indicate the performance of the ResNet-34 model and junior and senior nuclear medicine physicians, respectively. AUC, area under the curve. ROC, receiver operating characteristic curve. Compared to the performance of the junior and senior nuclear medicine physicians, the ResNet-34 model showed better diagnostic performance in identifying Graves’ disease, normality, subacute thyroiditis and thyroid tumors, with statistically significant differences between the model and physicians (**Supplementary Tables S1, S2**).

**Figure 5** illustrates the ROC curves for both the nuclear medicine physicians and the DCNN. The diagnostic performance of the junior and senior nuclear medicine physicians was compared with that of the DCNN. The model demonstrated significantly higher AUCs for all classes when classifying Graves’ disease, normal thyroid, subacute thyroiditis, and thyroid tumor SPECT images with both the internal and external validation sets. The AUCs of the model were significantly different from those of the two nuclear medicine physicians, with *p* < 0.05 in all cases (see **Supplementary Tables S1, S2**).

### DCNN model visualization

**Figure 6:**
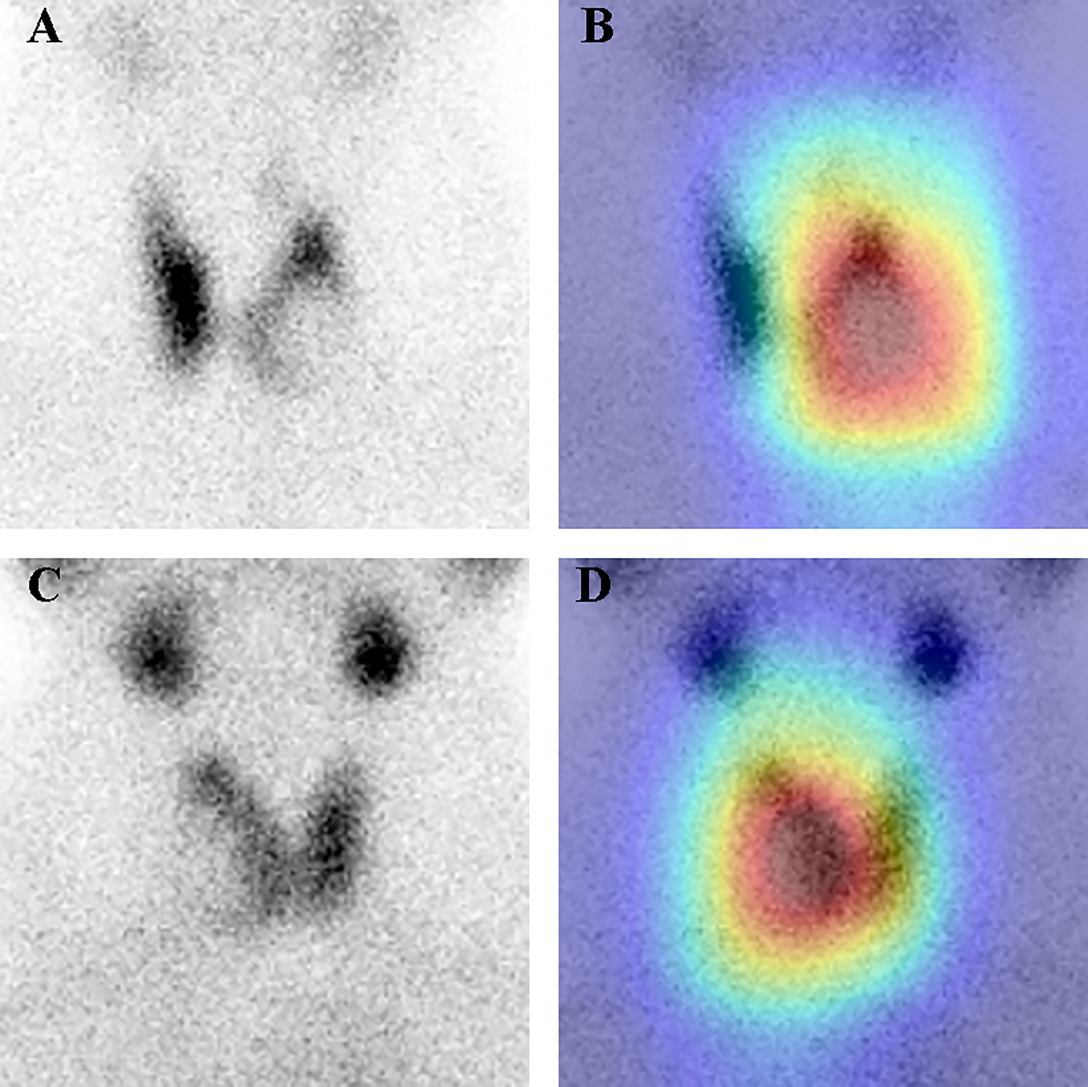
SPECT images and heatmaps of two clinical thyroid tumors. **(A, B)** Images from a 40-year-old male with a follicular adenoma cystic change on the left side of the thyroid. SPECT image **(A)** shows a normal thyroid position with a rounded radiolucent defect in the lower and middle left glandular shadow. The overlaid heatmap **(B)** is an example of a true positive case, where the deep learning model and the two nuclear medicine physicians correctly predicted a thyroid tumor. **(C, D)** A 46-year-old female with a follicular adenoma cystic change on the right side of the thyroid, with small papillae formation visible. Image **(C)** shows the thyroid gland positioned to the left. The right gland shadow has a posterolateral projection defect in the lower and middle parts. **(D)** A true positive result was predicted by a convolutional neural network model with an overlaid heatmap **(D)**. The junior physician made an incorrect prediction, and the experienced physician made a correct prediction.

During classification, the DCNN applied a final convolutional layer gradient to the thyroid tumor images for feature visualization during classification to generate heatmaps (**Figure 6**). The heatmaps for each DCNN model are shown in **Supplementary Figure S1**. The obtained heatmaps identify the region of thyroid tissue with the most typical features in the SPECT images, thus distinguishing this region from regions with normal thyroid tissue. The darker color of the heatmap indicates the greater contribution of the corresponding region of the original image to the network. Our findings demonstrate that the region of interest in the image using the improved ResNet-34 was broadly consistent with the actual lesion location, yielding the best performance.

## Discussion

Many studies have shown that computer-aided systems can be beneficial to clinicians in terms of improving their diagnostic skills (30–33). Our study confirms this by successfully using deep learning neural networks to build a model that intelligently identifies three thyroid diseases captured by SPECT. The results of our retrospective study indicate that the improved ResNet-34 model, which was evaluated with the internal validation dataset, achieved high accuracy, sensitivity, and specificity in the automated identification of Graves’ disease, subacute thyroiditis, and thyroid tumors in a real-world setting. After 200 epochs of training, the improved ResNet-34 model achieved accuracies of 0.961 and 0.931 for the internal and external validation sets, respectively, which were significantly higher than those of an experienced nuclear medicine physician (0.961 vs. 0.860, *p*<0.001; 0.931 vs. 0.868, *p*<0.001, respectively). To our knowledge, we are the first to apply deep learning to thyroid scintigraphy for the intelligent diagnosis of thyroid tumors.

Deep learning has garnered significant attention in recent years for its potential in diagnosing thyroid disorders. However, most models have focused exclusively on the benign and malignant classification of individual thyroid nodules, while other common disorders, such as hyperthyroidism, hypothyroidism, and thyroiditis, have received insufficient attention. Zhang et al. (30) addressed this gap by developing a deep learning model named the Hashimoto’s thyroiditis network (HTNet) that diagnoses Hashimoto’s thyroiditis using approximately 100,000 thyroid ultrasound images for training. HTNet showed a promising AUC of 0.905. In addition, Zhang et al. (15) performed multiple classifications of thyroid disease types using CT and ultrasound imaging modalities through the Xception model architecture. Although the model achieved high accuracy for identification with two thyroid datasets (0.942 and 0.972), no multicenter validation was performed. However, it is worth noting that the studies mentioned above only involved ultrasound and CT images and did not utilize the characteristic information from SPECT images to aid in diagnosis. In our study, the potential of DCNN-based deep learning models to assist clinicians in making efficient and accurate diagnoses by utilizing SPECT thyroid image features for disease and normal image screening tasks is demonstrated. Unlike human visual assessment, deep learning algorithms make final determinations based on the overall characteristics of thyroid SPECT images at different levels of radioactivity and across different locations. The advantage of deep learning algorithms is that they can consider the thyroid as a pixel-by-pixel volume in a classification task, providing a more accurate and quantitative assessment of thyroid imaging information compared to qualitative extrapolation. This allows for a more efficient and repeatable imaging diagnosis, reducing the risk of misdiagnosis due to manual subjective factors. Furthermore, the thyroid SPECT images utilized in our research were generated by different SPECT apparatuses, thereby augmenting the heterogeneity of data that can be employed to train the algorithm and assess the interpretation subjectivity among nuclear medicine physicians.

Although some studies have explored the use of AI for the automated classification of thyroid scintillation images, they all have certain limitations. We have summarized the distinctive aspects of our study compared to other studies (**Supplementary Table S3**). Qiao et al. (34) reported good diagnostic performance of deep learning models (i.e., AlexNet, VGG-16, and ResNet) in thyroid scintigraphy. Ma et al. (35) developed an optimized convolutional neural network for diagnosing thyroid diseases (i.e., Graves’ disease, subacute thyroiditis, and Hashimoto’s disease). Unlike the three-classification approach used by Qiao et al., both our study and Ma et al. employed a more comprehensive four-classification method. Additionally, our study incorporated thyroid tumor images, enabling clinicians to expedite the later-stage diagnosis and treatment of thyroid tumors, thus saving valuable time. In terms of accuracy, Qiao et al. achieved an overall accuracy of up to 86.8% in the classification of the three diseases, which is significantly lower than the diagnostic accuracy achieved in this study. It is noteworthy that although they compared DCNNs with clinicians, they did not perform multi-center validation or interpretability analysis. Similarly, while Ma et al. achieved higher accuracy, their study lacked comparison with clinicians and explanatory analysis, and relied solely on evaluation at a single hospital, limiting the generalizability of their findings. Consequently, our study stands out due to its larger dataset, more comprehensive methodology, and stronger model generalizability. Moreover, the DCNN model was able to diagnose over 500 images in the internal validation set in only 11 seconds, which was much more efficient than the performance of our nuclear medicine physicians. Overall, our model not only demonstrated consistent and superior accuracy in terms of classification performance compared to that of other DCNN models and physicians but was also more efficient in diagnosing diseases, allowing for more medical images to be processed, making it suitable for clinical applications.

The interpretability of current deep learning models is limited, making it challenging to understand how the algorithms process the input data and make internal connections to the final predicted labels (36). Therefore, it is crucial to ensure that during thyroid tumor identification, the DCNN model focuses on the SPECT features of thyroid tumors rather than irrelevant regions. To address this, we utilized the attentional heatmap generated by Grad-CAM to infer which part of the original input image the model focused on, thereby improving the model’s interpretability and providing additional confidence in the DCNNs’ classification ability. Furthermore, the activated areas superimposed on the original images can be used for qualitative assessment by nuclear medicine physicians in practice, enabling them to quickly check the points on which the classification is based.

Our study has several limitations that must be acknowledged. First, our study is a retrospective study, and the findings are based on the composition of a limited-sized dataset. Therefore, further extensive prospective studies are necessary to improve the findings before actual clinical application. Second, we collected typical images from patients with Graves’ disease, subacute thyroiditis, thyroid tumors, and normal thyroids retrospectively to train the model, and the number of images was limited. Third, in practice, only by analyzing all available images can a thorough and critical assessment of a patient’s thyroid be performed, and scans from the same patient may reveal features of numerous different diagnoses. However, in the proposed deep learning model, only one label is assigned to each image. Finally, although we performed external validation of images from two other hospitals in Chongqing and the improved ResNet-34 model performed well, the study was limited to within Chongqing. Thus, further multicity and multiregional studies are necessary to validate the results. We will consider all these limitations in our future scientific research to enhance the accuracy and efficiency of predicting thyroid diseases.

## Conclusions

The DCNN-based model proposed in this study has shown impressive results in accurately classifying various thyroid diseases, including Graves’ disease, subacute thyroiditis, thyroid tumors, and normal thyroid. In fact, the model performed better than experienced nuclear medicine physicians with both internal and external validation sets. Although the model is not always perfect, it can be used as a valuable tool to assist clinicians in making quick and precise diagnoses of thyroid disease in their daily practice. As the evidence supporting the potential of deep learning-based approaches continues to grow, clinicians may increasingly turn to AI-based diagnostic tools to reduce the time required for nuclear physicians to evaluate thyroid disease and to improve the accuracy of diagnosis.

## Data availability statement

The original contributions presented in the study are included in the article/**Supplementary Material**. Further inquiries can be directed to the corresponding authors.

## Ethics statement

The studies involving human participants were reviewed and approved by Ethics Committee of the Second Hospital of Chongqing Medical University. Written informed consent from the participants’ legal guardian/next of kin was not required to participate in this study in accordance with the national legislation and the institutional requirements. Written informed consent was obtained from the individual(s) for the publication of any potentially identifiable images or data included in this article.

## Author contributions

GY and HYZ designed the work. GY and HYZ contributed to conception and design of the study. CZ, WZ, and DF organized the database. WZ and YL performed the statistical analysis. HYZ and MR wrote the first draft of the manuscript. YL and CZ performed manual verification of image diagnosis. HHZ and JH completed the code together. GY carried out the manuscript correction. All authors contributed to the article and approved the submitted version.
