# Supplementary material for: Diagnosis of thyroid disease using deep convolutional neural network models applied to thyroid scintigraphy images: a multicenter study
Source: Front Endocrinol (Lausanne). 2023 Aug 11;14:1224191. doi: 10.3389/fendo.2023.1224191 (PMC10453808; doi:10.3389/fendo.2023.1224191)
Supplement: Supplementary file 1 [file DataSheet_1.pdf]

Electronic Supplementary Material

Description of the differences between the four thyroid image patterns

1. Graves' disease: This condition is characterized by an enlarged thyroid gland with a diffuse concentration of the imaging agent. The uptake and distribution of the agent are noticeably increased in the thyroid gland, while other tissues may appear decreased or absent, except for salivary glands.

2. Normal thyroid: A normal thyroid gland exhibits regular morphology and position of the lobes. The imaging agent is distributed evenly in both lobes, and the shadow of the bilateral submandibular glands appears fainter compared to the thyroid shadow.

3. Subacute thyroiditis: In cases of subacute thyroiditis, the thyroid glands exhibit ambiguous morphology with poorly defined contours. Early in the disease course, limited or diffuse radionuclide uptake is observed in most bilateral thyroid glands.

4. Thyroid tumors: Thyroid tumors can be either benign or malignant. Benign tumors are typically solitary nodules, with hot or warm nodules being more common. These adenomas grow slowly without invading or metastasizing to nearby organs. Malignant tumors manifest as solitary nodules, with cool or cold nodules being predominant. They have the potential to invade surrounding and contralateral normal tissues.

Data preprocessing

Image preprocessing was divided into three main steps: selecting the region of interest, data augmentation, and data normalization. The original SPECT thyroid images collected at hospitals contain a large amount of irrelevant information, such as acquisition time, physician markers and patient names, and regions. Appropriate interventions were required to prevent the model from learning useless information and wasting computational resources. First, the region of interest was selected for the image, irrelevant regions were cropped, and only the valuable information in the image was retained. The specific steps for the segmentation and cropping operation are as follows: The initial size of the image is 720 × 460 pixels. We select the shortest side, which is the width in this case, and use it as the side length to create a square image. Therefore, the final cropped image size is a × a pixels, where "a" represents the selected side length. By standardizing the image size, we can ensure it matches the input image size requirements of the model. The images were then resized to 256 × 256 pixels, a 224 × 224-pixel region was randomly cut out, the mean and variance of the training data were calculated, and then the training and validation data were normalized. Finally, the pixel values of grayscale images were made to lie in the range of 0 and 255 to induce a similar data distribution and obtain faster convergence of the deep learning algorithm. As thyroid SPECT images in our dataset were limited, to prevent the model from overfitting and improve the generalizability of the model, online data augmentation was performed on the images. Therefore, a series of data enhancement operations were performed during the model training, including random level flipping, random rotation, color dithering, adding random noise, and affine transformation. Finally, the image data were normalized as follows to improve the accuracy and generality of the models.

$$N = \frac{X - \mu}{\sigma}$$

X represents the original SPECT thyroid image, μ represents the mean pixel value, and σ denotes the standard deviation among all training images. Consequently, N reflects the normalized images used for network training. After data enhancement, all images were normalized so that the image pixel values were in the range of 0-255 to allow the model to converge faster during training.

The following is a specific description of the runtime environment.

Hardware:
- GPU: NVIDIA GTX 3080Ti
- Memory: 12GB

Software:
- Operating System: Linux 5.4.0
- Programming Language: Python 3.9
- Deep Learning Framework: PyTorch 1.10.1
- Data Processing Libraries: NumPy, Pandas
- Visualization Tool: Matplotlib

Model:
- Model Type: Deep Convolutional Neural Networks
- Dataset: Own dataset
- Image Resolution: 460×460

Table S1. The comparison of AUCs between ResNet34 and senior or junior nuclear medicine physician in internal validation.

AUC (internal dataset)	ResNet34 vs Junior	ResNet34 vs Senior	Senior vs Junior
Graves' disease	<0.001	=0.0181	<0.001
Normality	<0.001	<0.001	<0.001
SAT	<0.001	<0.001	=0.0115
Tumor	<0.001	<0.001	<0.001

Abbreviations: AUC area under the receiver operating characteristic curve; SAT subacute thyroiditis.

Table S2. The comparison of AUCs between ResNet34 and senior or junior nuclear medicine physician in external validation.

AUC (external dataset)	ResNet34 vs Junior	ResNet34 vs Senior	Senior vs Junior
Graves' disease	<0.001	=0.0166	<0.001
Normality	<0.001	<0.001	<0.001
SAT	<0.001	<0.001	<0.001
Tumor	<0.001	<0.001	<0.001

Abbreviations: AUC area under the receiver operating characteristic curve; SAT subacute thyroiditis.

Table S3. Comparative analysis with previous studies.

Method	Class	Accuracy	Compare with physicians	Multicenter validation	Attention heatmaps	Sample size
Qiao et al.	Graves' disease<br>SAT<br>Normality	86.8%	Yes	No	No	1430
Ma et al.	Graves' disease<br>SAT<br>Hashimoto's disease<br>Normality	99.3%	No	No	No	2888

This study.	Graves' disease<br>SAT<br>Tumor<br>Normality	94.8%	Yes	Yes	Yes	3194

Abbreviations: SAT, Subacute thyroiditis

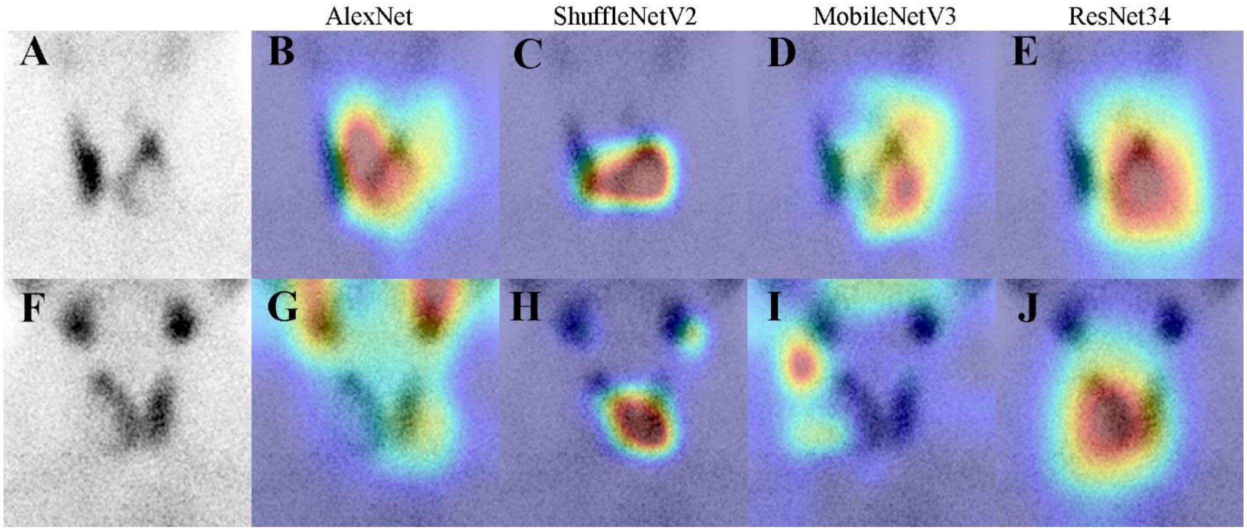

Figure S1. Comparison of Grad-Cam Heatmaps for Different DCNN Models. Representative thyroid tumor images and the corresponding Grad-Cam heat maps (Red and yellow areas represent areas of high activation and blue and green areas represent areas of low activation). a and f are the two original thyroid tumor images, and b-e and g-j are the gradient-weighted class activation mapping maps of the four DCNN models for images a and f, respectively.

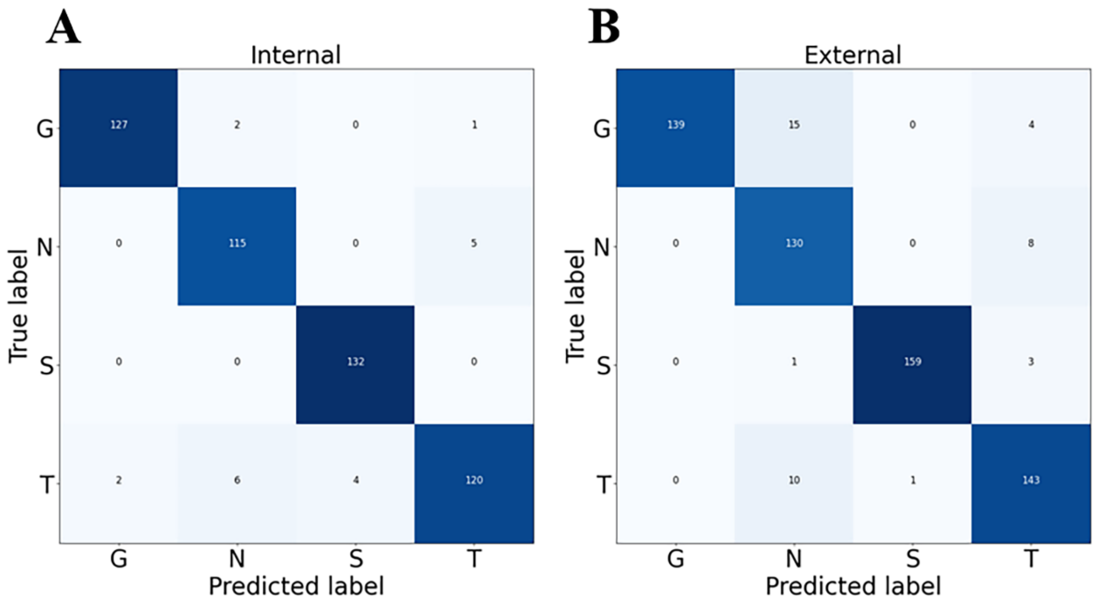

Figure S2. The confusion matrix of the improved ResNet34 in classifying four thyroid scintigrams in the internal and external validation. G: Graves' disease; N: normal thyroid; S: subacute thyroiditis; T: thyroid tumor.

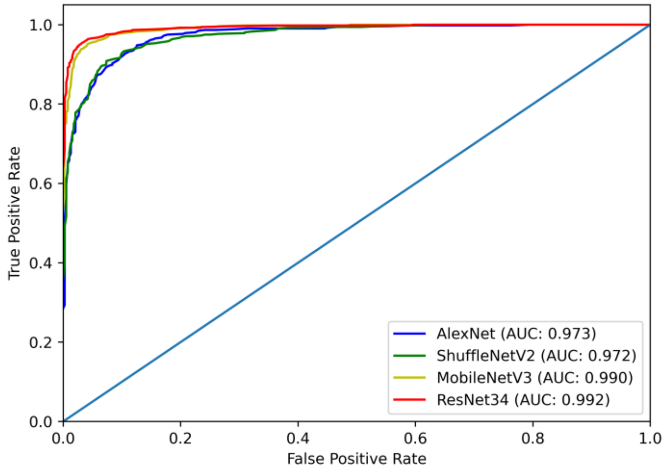

Figure S3. Receiver operating characteristic (ROC) curves of four DCNN models in the internal validation.
